# A functional CRISPR/Cas9 screen identifies kinases that modulate FGFR inhibitor response in gastric cancer

**DOI:** 10.1038/s41389-019-0145-z

**Published:** 2019-05-10

**Authors:** Jiamin Chen, John Bell, Billy T. Lau, Tyler Whittaker, Darren Stapleton, Hanlee P. Ji

**Affiliations:** 1 Division of Oncology, Department of Medicine, Stanford University School of Medicine, Stanford, CA 94305 USA; 2 Stanford Genome Technology Center, Stanford University School of Medicine, Stanford, CA 94305 USA

**Keywords:** Gastric cancer, Cancer genetics

## Abstract

Some gastric cancers have *FGFR2* amplifications, making them sensitive to FGFR inhibitors. However, cancer cells inevitably develop resistance despite initial response. The underlying resistance mechanism to FGFR inhibition is unclear. In this study, we applied a kinome-wide CRISPR/Cas9 screen to systematically identify kinases that are determinants of sensitivity to a potent FGFR inhibitor AZD4547 in KatoIII cells, a gastric cancer cell line with *FGFR2* amplification. In total, we identified 20 kinases, involved in ILK, SRC, and EGFR signaling pathways, as determinants that alter cell sensitivity to FGFR inhibition. We functionally validated the top negatively selected and positively selected kinases, ILK and CSK, from the CRISPR/Cas9 screen using RNA interference. We observed synergistic effects on KatoIII cells as well as three additional gastric cancer cell lines with *FGFR2* amplification when AZD4547 was combined with small molecular inhibitors Cpd22 and lapatinib targeting ILK and EGFR/HER2, respectively. Furthermore, we demonstrated that GSK3b is one of the downstream effectors of ILK upon FGFR inhibition. In summary, our study systematically evaluated the kinases and associated signaling pathways modulating cell response to FGFR inhibition, and for the first time, demonstrated that targeting ILK would enhance the effectiveness of AZD4547 treatment of gastric tumors with amplifications of *FGFR2*.

## Introduction

Gastric cancer is one of the leading causes of cancer-related deaths in the world^1^. In 2018, the American Cancer Society estimates that 26,240 cases will be diagnosed and 10,800 patients will succumb to gastric cancer in the United States^2^. Approximately 5–10% of gastric tumors harbor an amplification of the fibroblast growth factor receptor 2 (*FGFR2*) gene. This copy number alteration is associated with lymph node metastasis and poor prognosis^3^. FGFR signaling regulates a variety of crucial biological functions, such as cell proliferation, migration, differentiation, and cell death^4^. Human FGFRs are composed of four receptor tyrosine kinase receptors (FGFR1–4) as well as 18 FGF ligands. Gene amplification, activating mutations, and chromosomal translocations dysregulate the signaling of the members of the FGFR gene family. These somatic genetic alterations have been associated with tumorigenesis and progression in a range of cancers^4^. Cell lines and preclinical models have shown that *FGFR2* amplification is an essential driver in the development of gastric cancer^5^. Importantly, gastric cancer cells with high *FGFR2* amplification have an oncogenic dependency of FGFR signaling and are highly sensitive to the selective FGFR inhibitor AZD4547 both in vitro and in vivo^6^.

In a recent translational clinical trial, Turner and colleagues reported robust response to AZD4547 in gastric cancers with high *FGFR2* amplification^6^, suggesting that inhibition of FGFR signaling had potential as a targeted therapeutic. However, numerous clinical and experimental studies have demonstrated that tumors inevitably exhibit or develop drug resistance despite initial response to single agents, including FGFR2 inhibitors^7^. Therefore, elucidation of the underlying mechanisms of resistance to FGFR inhibition is critical to developing effective combinational therapies. There are several reports where long-term FGFR2-inhibitor exposure of sensitive *FGFR2* amplified gastric cancer cell lines and patient-derived xenograft (PDX) models lead to resistance^8–10^. However, there are no reported studies using systematic approaches to identify and characterize the determinants of sensitivity to FGFR inhibition.

High-throughput genomic screens, such RNA interference (RNAi) and clustered regularly interspaced short palindromic repeats CRISPR-associated nuclease Cas9 (CRISPR/Cas9), enable one to systematically perform loss-of-function screening in a wide range of biological processes and signaling pathways^11,12^. Compared with the traditional RNAi based gene perturbations, CRISPR/Cas9 knockout demonstrated superior on-target efficiency and minimum off-target effects^13^. In this study, we applied a kinome-wide CRISPR/Cas9 knockout assay to systematically investigate kinases as determinants of sensitivity to FGFR inhibition in KatoIII cells, a gastric cancer cell line with *FGFR2* amplification. We identified 20 candidate kinases that alter cell sensitivity, and confirmed that ILK, SRC, and EGFR signaling pathways have synergistic effects with FGFR inhibition. Moreover, we demonstrated that targeting ILK increased the effectiveness of FGFR inhibition for gastric cancer with *FGFR2* amplification.

## Results and discussion

### A Kinome-wide CRISPR/Cas9 screen identified kinases regulating cellular responses to FGFR2 inhibition

**Fig. 1:**
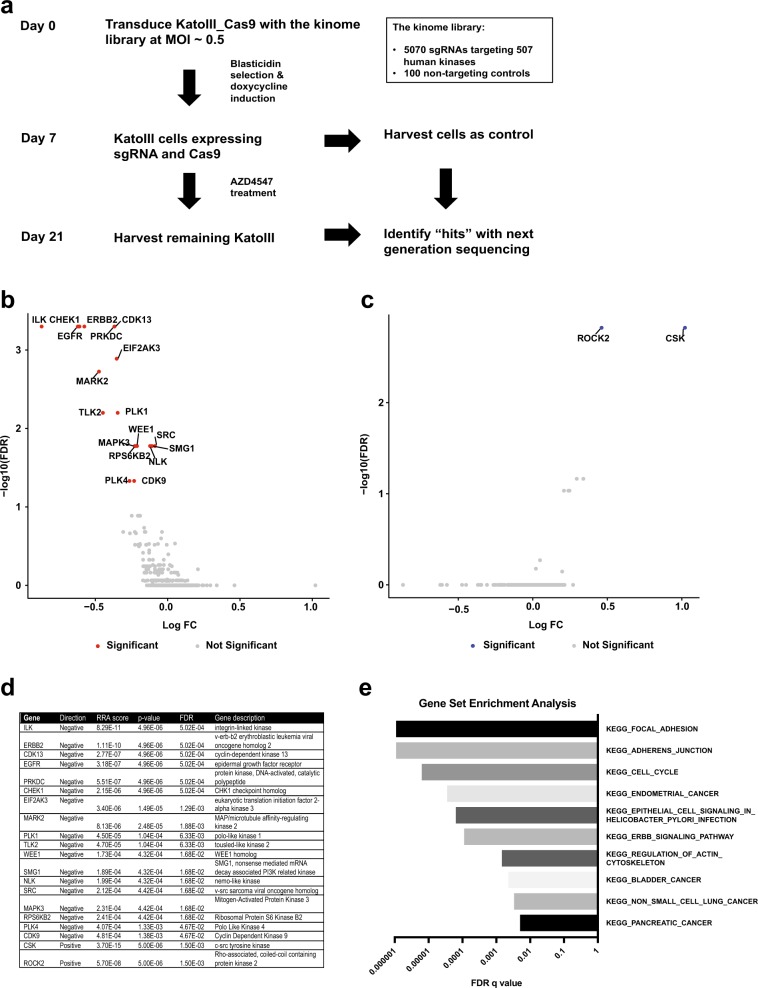
Kinome-wide CRISPR/Cas9 screen identified kinases regulating cellular responses upon FGFR inhibition. **a** Schematic overview of the experimental workflow. The whole procedure was repeated to generate two independent biological replicates. **b**, **c** Essential kinases identified by the knockout screen. Significant depleted (**b**) and enriched (**c**) kinases (FDR < 0.05) upon FGFR inhibition were labeled in red and blue, respectively. MAGeCK generates both negatively and positively selected false discovery rate (FDR) scores for each gene. Log FC: Log2 fold change. **d** The summary of essential kinases. Negatively enriched genes were assigned with values generated from negative selection and vice versa for positively enriched genes. RRA: robust ranking aggregation. **e** Top KEGG pathways identified by gene set enrichment analysis (GSEA) of the 20 essential kinases

Gastric cancer cells lines with *FGFR2* amplification, such as KatoIII and SNU16, are sensitive to AZD4547, a potent small molecular FGFR1-3 inhibitor^5,14^, while gastric cancer cells lines with no *FGFR2* amplification, such as AGS and SNU16, are insensitive to AZD4547 (Supplementary Fig. S1). However, despite the IC_50_ being <10 nM, we often observed that ~15–20% of KatoIII and SNU16 cells are viable after exposure to 100 nM AZD4547 (Supplementary Fig. S1). Emerging studies have demonstrated the genetic and transcriptional heterogeneity within the cancer cell lines, resulting in varied drug responses to targeted therapies^15,16^. We speculated that the heterogeneity within tumors with *FGFR2* amplification would be clinically manifested as residual tumor cells, leading to relapses in single agent AZD4547 treatment. For example, studies have reported tumor regrowth after tumor regression during AZD4547 treatment interval in patient-derived gastric cancer mouse xenograft models harboring FGFR2 amplification^5,8^. To identify druggable kinase targets that can increase the efficacy of AZD4547 and reduce the resistance, we applied a kinome-wide lentiviral CRISPR/Cas9 knockout screen to identify kinases that modulate the cellular sensitivity upon FGFR inhibition (Fig. 1a). The kinome-wide lentiviral library included 5070 sgRNAs targeting 507 human kinases and 100 non-targeting control sgRNAs^17^. We sequenced the plasmid library pool and confirmed the sgRNA representation and pool complexity with ~6-fold change of the abundance between the 10th and 90th percentiles (Supplementary Fig. S2). We established a doxycycline-inducible Cas9 expressing KatoIII cells (KatoIII_Cas9), and the expression of Cas9 nuclease upon doxycycline treatment was confirmed by western blot (Supplementary Fig. S3). Transduced with the lentivirus pool at Day 0, the KatoIII_Cas9 cells were subsequently induced by doxycycline and selected with Blasticidin from Day 2. At Day 7, 6 million cells were saved as control and 24 million cells were treated with 100 nM AZD4547 for another 14 days before harvesting the remaining cells. The CRISPR/Cas9 screen was performed twice to generate biological replicate samples, which were subsequently deep sequenced to determine the abundance of each sgRNA (Supplementary Table S1). The methods for the CRISPR/Cas9 library preparation, screen, and data analysis were described in the supplementary materials.

To identify essential kinases, specifically negatively and positively selected genes upon FGFR2 inhibition, we applied the model-based analysis of genome-wide CRISPR/Cas9 knockout (MAGeCK) and the visualization framework (VISPR) algorithms to analyze the read counts of each sgRNA^18,19^. The normalized read count distribution demonstrated the consistency between samples and the pairwise Pearson correlation displayed strong coefficients (*r* ≥ 0.93) between log read counts (Supplementary Fig. S4a-b). The negatively (or positively) selected sgRNA were predicted to target kinases as loss-of-function of which would decrease (or increase) the viability of KatoIII cells upon FGFR2 inhibition (Supplementary Fig. S5 and Supplementary Table S2). With the false discovery rate (FDR) < 5%, we identified 20 kinases altering the cell responses upon FGFR2 inhibition, including 18 kinases were negatively selected and 2 kinases were positively selected (Fig. 1b–d and Supplementary Table S3). Hahn and colleagues have reported that sgRNAs targeting genomic amplifications in cancer cells may elicit a gene-independent antiproliferative cell responses^20^, which might lead to false identification of essential sgRNAs during the knockout screen. We examined the genomic area of negatively selected kinase genes in KatoIII and confirmed that none of the 18 genes were located at genomic amplified region. Pathway analysis based on gene set enrichment analysis (GSEA) predicts that candidate kinases are enriched in KEGG pathways involved in cell adhesion (focal adhesion and Adherens junction), cell cycle, and ErbB (aka EGFR) signaling (Fig. 1e).

The integrin linked kinase gene (*ILK*) was the top ranked negatively selected kinase. ILK functions as a scaffold protein by connecting integrins to the actin cytoskeleton and also acts as kinase involved in a variety of signaling pathways^21^. ILK has emerged as a potential therapeutic target in cancer based on previous findings that overexpression of ILK promotes cancer cell survival and invasion, while inhibition of ILK induces cell cycle arrest and apoptosis^22^. EGFR and ERBB2, members of epidermal growth factor receptor (EGFR) family, were also present in the negatively selected kinases. This result is supported by a previous finding that AZD4547 and EGFR inhibitor synergistically inhibited the growth of gastric cancer cells with *FGFR2* amplification^10^. On the other hand, activation of FGFR signaling was reported to promote resistance to ERBB2 inhibition^23^, suggesting signaling compensation between EGFR and FGFR pathway activities.

C-terminal Src kinase (*CSK*) and Rho-associated protein kinase 2 (*ROCK2*) were the only two positively enriched kinase genes in our knockout screen. Of significant relevance, CSK functions as a master negative regulator of SRC family kinases. SRC is among the negatively selected kinases, suggesting activation of SRC signaling would attenuate the inhibitory effects of AZD4547. Therefore, knocking out CSK may lead to the activation of SRC family kinases and downstream pathways, subsequently modulating cells less sensitive to FGFR2 inhibition. In summary, our screen result identified candidate kinases, i.e., ILK, SRC, and EGFRs, that would modulate cellular response to FGFR2 inhibition.

### siRNA knockdown confirmed the CRISPR screening results

**Fig. 2:**
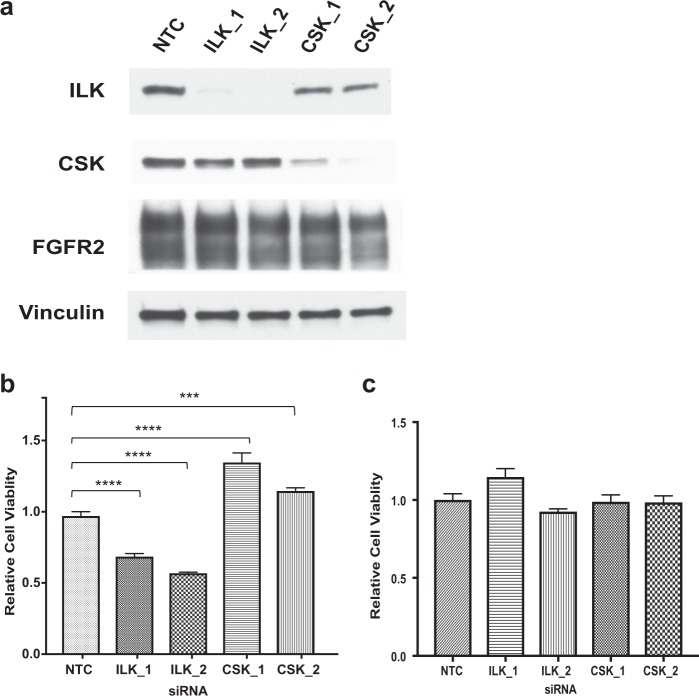
siRNA validation of ILK and CSK as kinases modulate sensitivity to FGFR inhibition. **a** Western blot confirmed that ILK and CSK gene expression was effectively knocked down in KatoIII cells. Cells were transfected with 10 nmol/l oligos and were harvested 72 h. ILK (#3856) and CSK (#4980) antibodies were obtained from Cell Signaling. Vinculin (#13901, Cell Signaling) was the loading control. **b** Upon AZD4547 treatment, knocking down ILK decreased cell viability while knocking down CSK increased cell viability. **c** Knocking down ILK or CSK alone does not affect KatoIII cell viability. KatoIII cells were seeded in 6-well plates at 300,000 cells/well the day before transfection and were transfected with 10 nM siRNA oligos using LipoJet (SignaGen Laboratories). The Dicer-substrate short interfering RNAs (DsiRNAs) targeting CSK (CSK_1 Reference #146599035 and CSK_2 #146599032), ILK (ILK_1 Reference #146599024 and ILK_2 #146599021) as well negative control oligos (NTC) were obtained from IDT. The viability was measured 6 days by CellTiter-Glo after treated with AZD4547 or plain median. Representative data from one of three independent experiments are shown. The data are measured in hextuple as mean ± s.e.m. ****P < 0.0001; **P < 0.01. The *P* values were calculated by Dunnett’s multiple comparisons test

We verified the role of the two top candidate kinases identified by CRISPR/Cas9 knockout screen: ILK (negatively selected) and CSK (positively selected). We applied short interfering RNA (siRNA) to knockdown candidate genes in KatoIII cells upon FGFR2 inhibition. We evaluated the transfection efficiency of KatoIII cells and determined that > 95% of KatoIII cells were transfected with siRNAs (Supplementary Fig. S6). Western blot demonstrated the level of ILK and CSK expression were drastically reduced after siRNA transfection (Fig. 2a). Knocking down ILK significantly increased the sensitivity of KatoIII to AZD4547 while knocking down CSK led to the opposite effect (Fig. 2b), confirming the CRISPR/Cas9 screen results. Importantly, knocking down ILK or CSK alone did not change KatoIII cell viability (Fig. 2c) while knocking down FGFR2 using siRNAs significantly reduced KatoIII cell viability (Supplementary Fig. S7). Our results indicated that ILK and CSK were involved in pathways specifically modulating cellular responses upon FGFR inhibition.

### ILK and EGFR inhibitors increase sensitivity to AZD4547 in *FGFR2* amplified gastric cancer cells

**Fig. 3:**
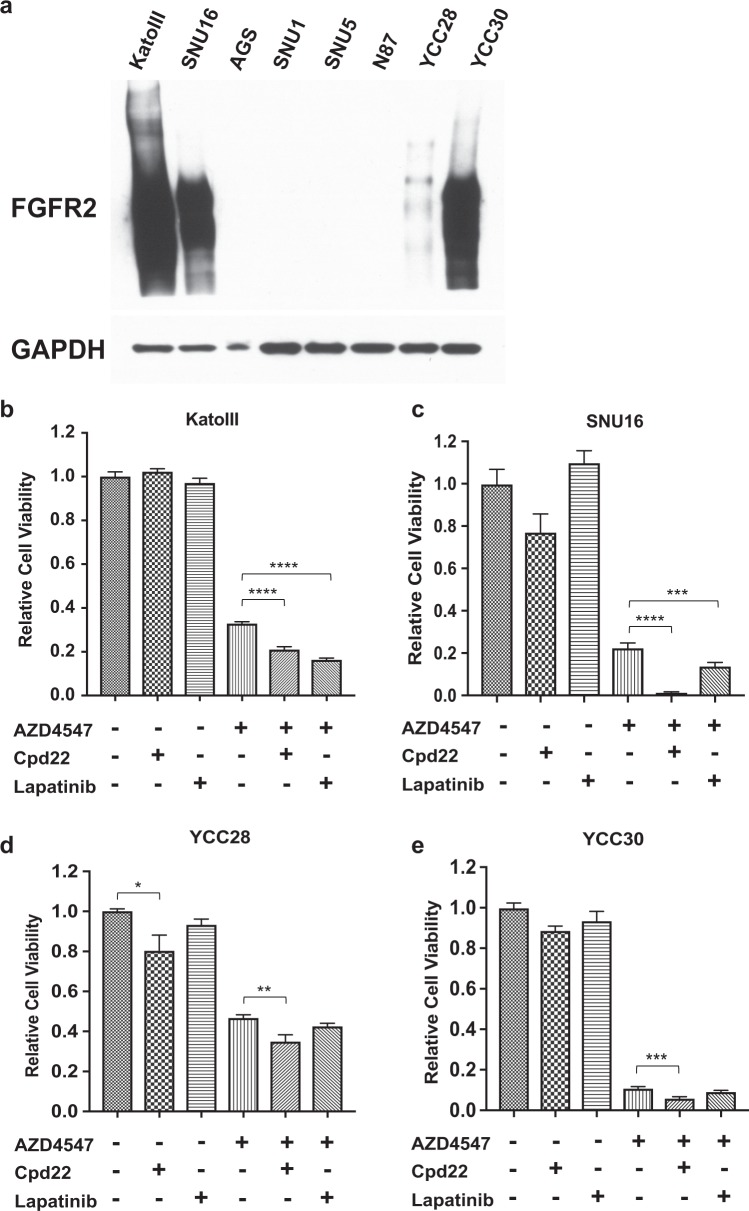
Pharmacological inhibition of ILK and EGFR signaling confirmed the synergistic effects with FGFR inhibition. Overexpression of FGFR2 was detected in KatoIII, SNU16, YCC28, and YCC30 cells by western blot. FGFR2 (#11835) and GAPDH (#5174) antibodies were purchased from Cell Signaling. Gastric cancer cell lines KatoIII and SNU16 were purchased from ATCC, and YCC-28 and YCC-30 were kindly provided by Dr. Sun Young Rha at Song-dang Institute for Cancer Research (Yonsei University, Seoul, Korea). Cells were cultured in IMDM supplemented with 20% FBS or RPMI-1640 supplemented with 10% FBS. Cell viability of KatoIII (**b**), SNU16 (**c**), YCC28(**d**), and YCC30 (**e**) were measured by CellTiter-Glo after treated with Cpd22 and lapatinib alone or in combination with AZD4547 for 96 h. The data are measured in hextuple or quintuple and displayed as mean ± s.e.m. ****P < 0.0001; ***P < 0.001; **P < 0.01; *P < 0.05. The *P* values were calculated by Dunnett’s multiple comparisons test

A wide repertoire of small molecular inhibitors are available for inhibiting specific kinases^24^. To evaluate the potential of a combinatorial strategy, we identified the molecule Cpd22 that inhibits ILK activity and drug lapatinib that inhibits ERBB2/EGFR signaling amplification. We determined whether these inhibitors have synergistic effects when combined with AZD4547 in gastric cancer cells with *FGFR2* overexpression. We assessed the activity of drug combinations using three more gastric cancer cell lines (SNU16, YCC28 and YCC30) with various degrees of FGFR2 overexpression in addition to KatoIII cells (Fig. 3a).

KatoIII displayed little sensitivity to Cpd22 or Lapatinib alone at the concentrations that inhibit ILK (Cpd22: 2 µM)^25^ and ERBB2/EGFR (Lapatinib: 10 µM)^26^ in vitro, respectively (Fig. 3b). When combined with AZD4547, both Cpd22 and Lapatinib significantly enhanced KatoIII’s sensitivity to AZD4547 (Fig. 3b), confirming ILK and EGFR signaling pathways could modulate cellular responses to FGFR2 inhibition. Similar to KatoIII cells results, SNU16, YCC28, and YCC30 displayed varied degree of sensitive to AZD4547, but little or no sensitivity to Cpd22 or Lapatinib alone. Importantly, Cpd22 significantly decreased cell viability when combined with AZD4547 (Fig. 3c–e). We also noticed the significant killing effects when Lapatinib is combined with AZD4547 in SNU16, though the effects were less notable in YCC28 and YCC30. Furthermore, we performed dosage combinations of AZD4547 and Cpd22 in KatoIII and SNU16 using the the Chou-Talalay method^27^. The combination index (CI) was <1 when AZD4547 and Cpd22 were combined at varying doses (Supplementary Table S4), indicating the effect is synergistic. To examine the specificity of Cpd22 on ILK, KatoIII cells with ILK knockdown was treated with both of AZD4547 and Cpd22. As expected, Cpd22 in combination with AZD4547 notably eliminated the effects of ILK knockdown on KatoIII (Supplementary Fig. S8) when compared with the AZD4547 treatment alone described previously (Fig. 2b). In summary, our data demonstrated that ILK inhibitor and to lesser extent EGFR inhibitor could further sensitize *FGFR2* amplified gastric cancer cells to FGFR inhibition.

### GSK3b is a downstream target of ILK upon FGFR inhibition

ILK mediates a variety of cell signaling pathways, including phosphatidylinositol-3-kinase (PI3K)-AKT, MAPK, Wnt, Transforming growth factor (TGF)-β, and Hippo signaling in cancer cells^28–30^. Overexpression of ILK promotes cancer cell survival and is associated with a variety of cancers, including prostate, ovarian, breast, pancreatic, melanoma, colon and gastric tumors^22,31,32^. Yasui and colleagues reported the expression of ILK protein in the primary gastric tumor samples but not in the normal gastric tissues^33^, suggesting *ILK* was associated with gastric tumor development. We examined *ILK* status in the 415 stomach adenocarcinomas using genomic data from the Cancer Genome Atlas (TCGA). *ILK* had upregulated gene expression in the 16 tumors (4%) based on RNA sequencing and was mutated (missense) in 10 tumors (2%) based on whole-exome sequencing (Supplementary Fig. S9). No amplification or deletion of *ILK* gene was detected. Kaplan–Meier analysis indicated that patients with *ILK* upregulation tend to have lower survival time (median = 26.08 months) compared with patients without *ILK* upregulation (median = 34.26 months). However, the difference was not significant (Logrank test *P*-value = 0.203), which is, at least in part, likely due to fewer cases with *ILK* upregulation.

**Fig. 4:**
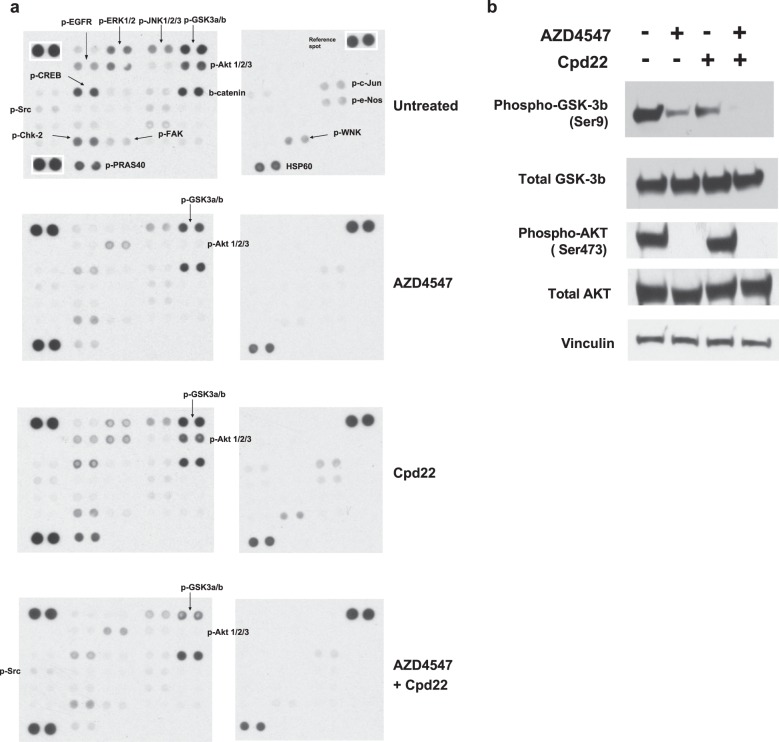
GSK-3b is a downstream target of ILK upon FGFR inhibition. **a** KatoIII cells treated with vehicle control, AZD4547, Cpd22, or AZD4547/Cpd22 for 1 h. Four hundred micrograms of cell lysates were incubated with membrane to detect phosphorylation of 43 kinases and two total proteins using human phospho-kinase array (R&D Systems) according to manufacturer’s instruction. Each kinase was detected in duplicate. Reference spots labeled with the white box. **b** Western blot of the cell lysates used in (**a**), probed for p-GSK-3b (Ser9), total GSK-3b, p-AKT (Ser473), and total AKT. Vinculin is used as the loading control

To identify the downstream effectors of ILK upon FGFR inhibition, we assessed the phosphorylation of 43 kinases in KatoIII cells treated with AZD4547 and Cpd22, alone or with a combination of both using phospho-kinase antibody arrays. We noticed a global reduction of protein phosphorylation in KatoIII cells after AZD4547 treatment (Fig. 4a). Dephosphorylation at kinases, e.g., ERK1/2, JNK1/2/3, Glycogen synthase kinase 3 (GSK-3), AKT1/2/3, focal adhesion kinase (FAK), SRC, Chk-2, WNK, and e-NOS, indicated that FGFR inhibition repressed a wide range of signaling pathways, including MAPK, PI3K-AKT, FAK-SRC, and vascular endothelial growth factor (VEGF) signaling^34^. The FGFR inhibition did not affect the total protein expression of b-catenin and HSP60. Inhibition of ILK using Cpd22 also reduced the expression of phosphorylated kinases, though to a lesser extent compared with AZD4547 (Fig. 4a). Combination of AZD4547 and Cpd22 globally decreased kinase phosphorylation, which is similar to what we observed from using only AZD4547. Notably, we observed further reduction of phosphorylation at p-GSK3a/b (Ser9/Ser21) compared with AZD4547 and Cpd22 treatment alone. Studies have demonstrated that the p-GSK3b at Ser9 and p-Akt at Ser473 were direct substrates of ILK in various cell types^29^. Our data demonstrated that FGFR inhibition alone inhibited p-Akt at Ser473, but was not sufficient to inhibit p-GSK3a/b (Ser21/Ser9). Using western blotting, we confirmed that combination of AZD4547 and Cpd22 greatly reduced the p-GSK3b at Ser9 compared with treatment with AZD4547 and Cpd22 alone, while the expression of total GSK3b remained unchanged (Fig. 4b). As expected, FGFR inhibition alone was sufficient to inhibit p-Akt at Ser473 (Fig. 4b). In summary, our data suggested that p-GSK3b (Ser9) was likely to be one of the downstream effectors of ILK signaling upon FGFR inhibition.

Two isoforms of GSK, GSK3a and GSK3b, often negatively regulate target proteins by promoting protein ubiquitination and degradation^35^. GSK3 regulates a variety of biological processes, including metabolism, proliferation, and survival. Pro-survival PI3K-AKT signaling pathways inactivate GSK3 activity by phosphorylating its N-terminus, i.e., GSK3b at Ser9 and GSK3a at Ser21^36^. Our results demonstrated that inhibition of FGFR signaling greatly reduced the PI3K-AKT signaling by dephosphorylating p-AKT, but only had moderate effect on p-GSK3 (Fig. 4). When both FGFR and ILK signaling pathways were inhibited, we observed a significant reduction of p-GSK3 level, which ultimately lead to more active GSK3 proteins. GSK3 negatively regulate cell cycles by targeting MYC, cyclin D, and cyclin E for degradation^35^. Furthermore, studies have demonstrated that GSK3b promotes apoptosis through the mitochondria-mediated intrinsic apoptotic pathways upon inhibition of the PI3K-AKT signaling pathways^37^. For example, GSK3b transforms pro-apoptotic protein Bax to an active state^38^ and mediates the destabilization of anti-apoptotic protein Mcl-1 by ubiquitination^39^. Therefore, inhibition of ILK would activate GSK3b activity by reducing p-GSK3b (Ser9), resulting in further sensitization of cancer cells to FGFR inhibition. So and colleagues reported an AKT-independent PKC-mediated phosphorylation of GSK3b mechanism that increases the resistance to FGFR inhibitor in a PDX model^8^. Taken together, our studies provide evidence that reactivation of GSK3b by targeting GSK3b phosphorylation may reduce the resistance and enhance the effectiveness of FGFR inhibitors.

## Supplementary information

Supplementary methods

Supplementary Figures

Supplementary tables
