# Supplementary material for: A functional CRISPR/Cas9 screen identifies kinases that modulate FGFR inhibitor response in gastric cancer
Source: Oncogenesis. 2019 May 10;8(5):33. doi: 10.1038/s41389-019-0145-z (PMC6510732; doi:10.1038/s41389-019-0145-z)
Supplement: Supplementary file 1 — Supplementary methods [file 41389_2019_145_MOESM1_ESM.docx]

**Supplementary Methods and Results**

**A functional CRISPR/Cas9 screen identifies kinases that modulate FGFR2 inhibitor response in gastric cancer**

Jiamin Chen^1^, John Bell^2^, Billy T Lau^2^, Tyler Whittaker^1^, Darren Stapleton^1^, Darren Stapleton^1^, Hanlee P Ji^1,2^

^1^Division of Oncology, Department of Medicine, Stanford University School of Medicine

^2^Stanford Genome Technology Center, Stanford University School of Medicine

*To whom correspondence should be addressed.

Hanlee P. Ji

Email: genomics_ji@stanford.edu

Phone: 650-721-1503

Fax: 650-725-1420

**Supplementary Methods**

**Creation of a Dox-inducible Cas9 expressing KatoIII cell line**

Cas9 lentivirus was generated from HEK-293T cell transfected with pCW-Cas9 plasmids using the standard lentivirus production protocol. The Dox-inducible Cas9 expressing pCW-Cas9 was a gift from Eric Lander & David Sabatini (Addgene plasmid # 50661). KatoIII cells were transduced by Cas9 lentiviral particles followed by puromycin selection (2ug/ml) 24h after infection. After selection, individual KatoIII_Cas9 cells were sorted into 96-well plates, and cultured under puromycin selection for 4 weeks. Individual colonies were replated into 12-well plates.

**Propagation of Kinome sgRNA libraries and Lentivirus production**

Human kinase (Addgene # 51044) and Control sgRNA libraries (Addgene #51048) were gifts from David Sabatini & Eric Lander. The Human kinase sgRNA pool includes 5070 sgRNAs targeting 507 kinase genes and the control sgRNA pool includes 100 non-targeting sgRNAs (Supplementary table S1) [11]. The procedure of amplifying pooled sgRNA library was based on the protocol described previously [37].

The lentiviral CRISPR library was produced by transfecting the combined Kinase sgRNA plasmid pool and Control plasmid sgRNA pool (total sgRNAs = 5170) with packaging plasmids pMD2.G and psPAX2 in three T75 flasks of HEK-293T cells using standard lentivirus production protocol. The viruses were harvested at 48h and 72h post-transfection.

**Whole Kinome CRISPR screening in AZD4547 treated KatoIII cells**

Spin-infected ~20 million KatoIII_cas9 cells with the kinome CRISPR lentivirus library at MOI =0.5 in 12-well plates at Day 0, and transferred the cells into four T75 flasks at Day 1. Blasticidin selection (10 ug/ml) and doxycycline induction (1ug/ml) started at Day 2, and cells were harvested at Day 7. Six million cells were saved as control, and 24 million cells were treated with 100 nM AZD4547 for another 14 days before harvesting the remaining cells (~ six million cells). The whole procedure was performed twice and generated four biological samples.

The genomic DNA (gDNA) was extracted from control and AZD4547 treated cells using the Maxwell® 16 Cell DNA Purification Kit (Promega). The sgRNA sequences were amplified by a nested PCR. In order to achieve 300X coverage of 5170 sgRNAs and assuming individual cell contains 6.6 pg gDNA, 10 ug of input gDNA (6.6 pg X 5170 X 300 = 10 ug) from each sample was used in the first PCR. A total of 20 PCR reactions were performed using the Q5 High-Fidelity DNA Polymerase (NEB). The primers for the first PCR (18 cycles, product size = 477):

OutF: GCCGGCTCGAGTGTACAAAA

OutR: AGCGCTAGCTAATGCCAACT.

10ul of the first PCR product was used for the second PCR. The primers for the second PCR (22 cycles, product size = 239):

In-F: AATGATACGGCGACCACCGAGATCTACACTCTTTCCCTACACGACGCTCTTCCGAT

CTTCTTGTGGAAAGGACGAAACACC

In_R1: CAAGCAGAAGACGGCATACGAGAT*ATCACG*GTGACTGGAGTTCAGACGTGTGCTCT

TCCGATCTACCGACTCGGTGCCACTTTT

In_R2: CAAGCAGAAGACGGCATACGAGAT*CGATGT*GTGACTGGAGTTCAGACGTGTGCT

CTTCCGATCTACCGACTCGGTGCCACTTTT

ln_R3: CAAGCAGAAGACGGCATACGAGAT*TTAGGC*GTGACTGGAGTTCAGACGTGTGC

TCTTCCGATCTACCGACTCGGTGCCACTTTT

ln_R4: CAAGCAGAAGACGGCATACGAGAT*TGACCA*GTGACTGGAGTTCAGACGTGTGC

TCTTCCGATCTACCGACTCGGTGCCACTTTT

The reverse primers included a six-nucleotide barcode, italicized and underlined, to multiplex the biological samples. The final PCR products were pooled, purified and size selected using AMPure XP Beads (Beckman Coulter), and eventually sequenced using a NextSeq 500/550 Mid Output v2 kit (300 cycles) with 50% PhiX spike-in (Illumina).

**Data analysis**

The 20 nt sgRNA sequences were extracted from the FASTQ files generated by the sequencer using a python script, and then aligned with bowtie2 against the library of kinome sgRNAs. After being sorted, the 20mers were filtered to only include cases that had no indels (correct alignment flag, alignment position = 1, CIGAR string = 20M), although mismatches were tolerated. The number of reads aligning against each kinase sequence was counted up using command line tools and then associated with the kinase sequence name using a python script.

The positively and negatively selected genes were ranked and visualized using the algorithm MAGeCK-VISPR [16, 17]. MAGeCK ranks sgRNAs based on P-values calculated from a negative binomial model, and uses a modified robust ranking aggregation algorithm to identify positively or negatively selected genes.
