# Supplementary material for: A functional CRISPR/Cas9 screen identifies kinases that modulate FGFR inhibitor response in gastric cancer
Source: Oncogenesis. 2019 May 10;8(5):33. doi: 10.1038/s41389-019-0145-z (PMC6510732; doi:10.1038/s41389-019-0145-z)
Supplement: Supplementary file 2 — Supplementary Figures [file 41389_2019_145_MOESM2_ESM.pdf]

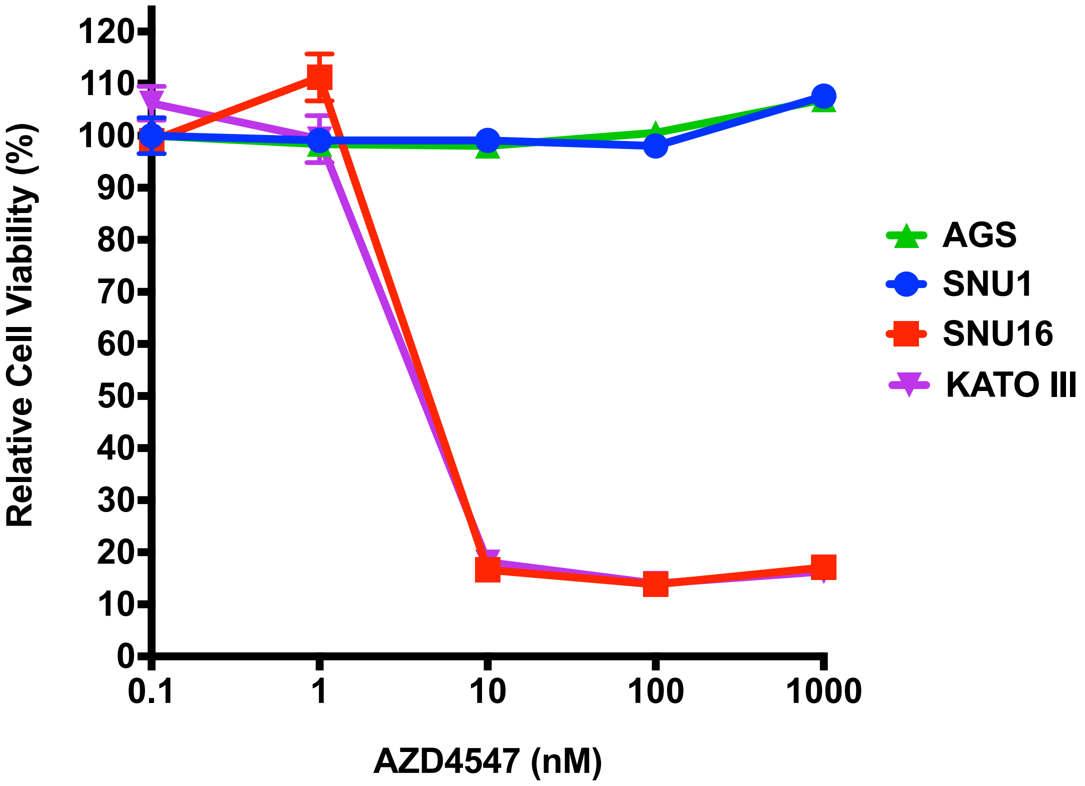

Figure S1. The viability of gastric cancer cells to targeted FGFR inhibition. Cells were seeded in 96-cell plates and treated with FGFR inhibitor AZD4547 for 96h. The cell viability was measured by CellTiter-Glo in hextuple or quintuple and displayed as mean±s.e.m.

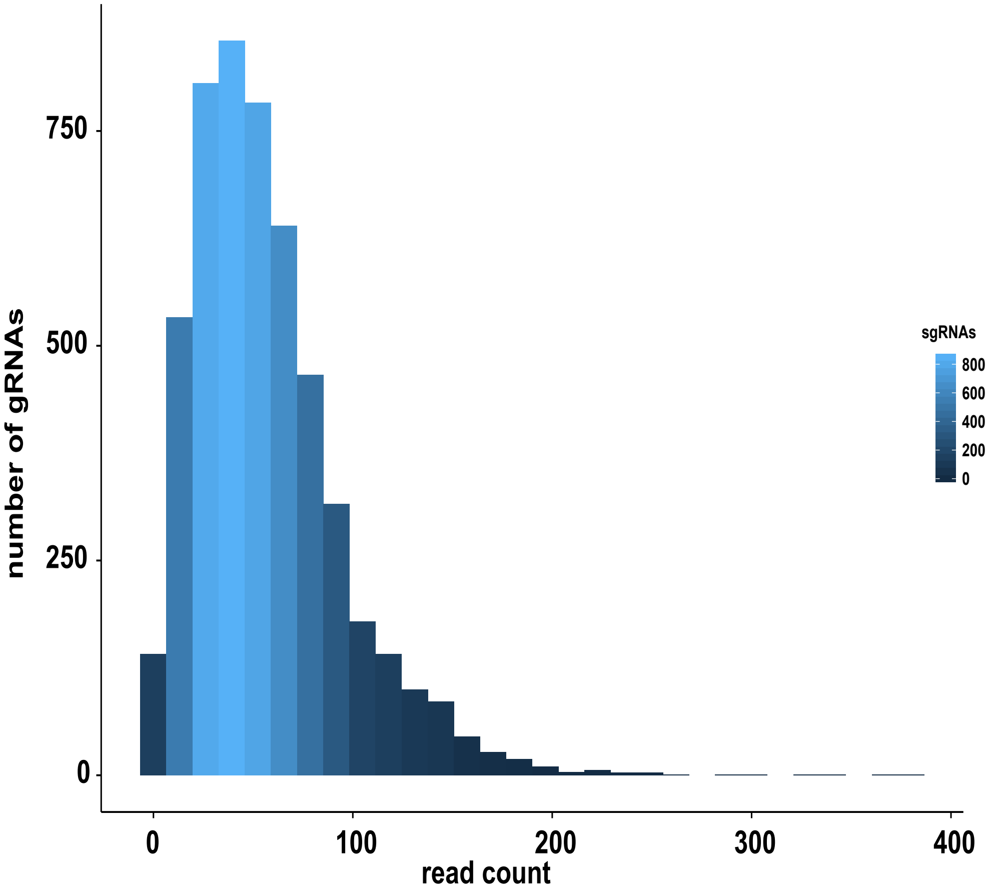

sgRNA #	5170
Total Counts	294073
Medium	49
1th Percentile	3
10th Percentile	17
90th Percentile	106

Figure S2. Overview of sgRNA distribution in the amplified plasmid library. 2ul from sgRNA library was transformed into 25 ul of Endura electrocompetent cells (Lucigen) by electroporation and plated on five ampicillin dishes (100 mm) overnight at 32C. The bacteria colonies were scraped off from the plates, and plasmid DNA was extracted using the HiPure Plasmid Maxiprep Kit (Invitrogen). The sgRNA library representation was confirmed by targeted sequencing using a MiSeq Reagent Nano Kit v2 (300-cycles, Illumina). The primers to directly amplify the sgRNA sequences on the pLX-sgRNA plasmids (product size = 239):

Ln_F:AATGATACGGCGACCACCGAGATCTACACTCTTTCCCTACACGACGCTCTTCC GATCTTCTTGTGGAAAGGACGAAACACC

Ln_R:CAAGCAGAAGACGGCATACGAGATTTAGGCGTGACTGGAGTTCAGACGTGTG CTCTTCCGATCTACCGACTCGGTGCCACTTTT

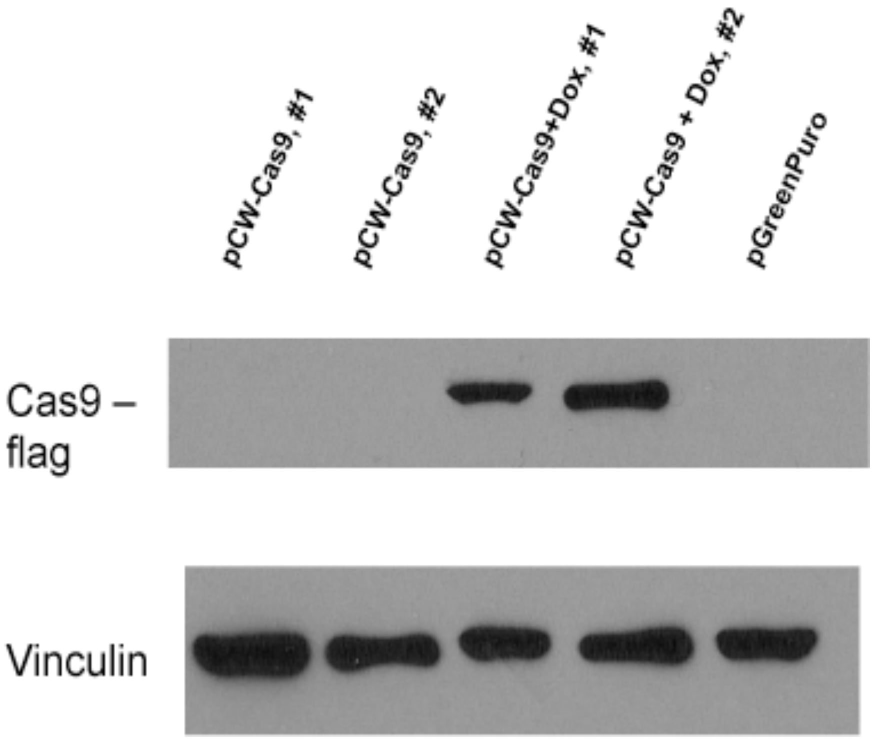

Figure S3. KatoIII_Cas9 cells express Cas9 upon doxycycline induction. The clones of KatoIII_Cas9 cells were induced with doxycycline (1 ug/ml) for 48h. Cas9 protein was tagged with FLAG, and anti-FLAG (F3165, Sigma-Aldrich) was used to detect Cas9 expression.

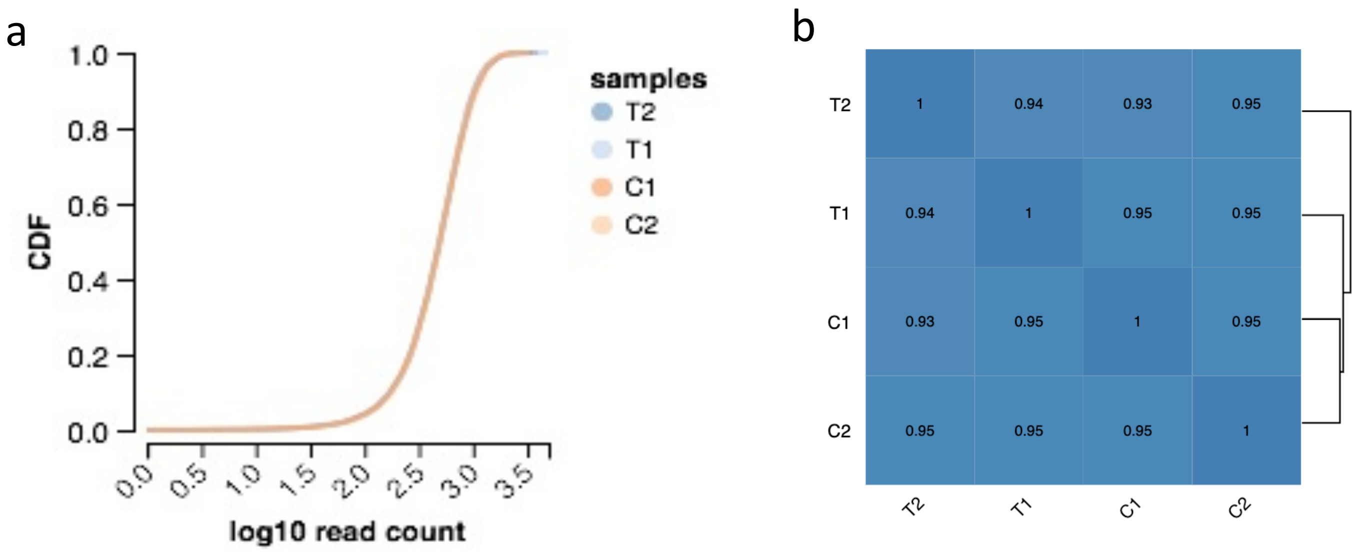

Figure S4. The quality control measurements for the whole-kinome knockout screen. (a) Cumulative distribution of normalized read counts between samples. (b) Pairwise Pearson correlations between sample log read counts. The KatoIII cells under screen before (C1- Control 1; C2 - Control 2) and after AZD4547 treatment (T1 – treatment 1; T2 – treatment 2). CDF - cumulative distribution function.

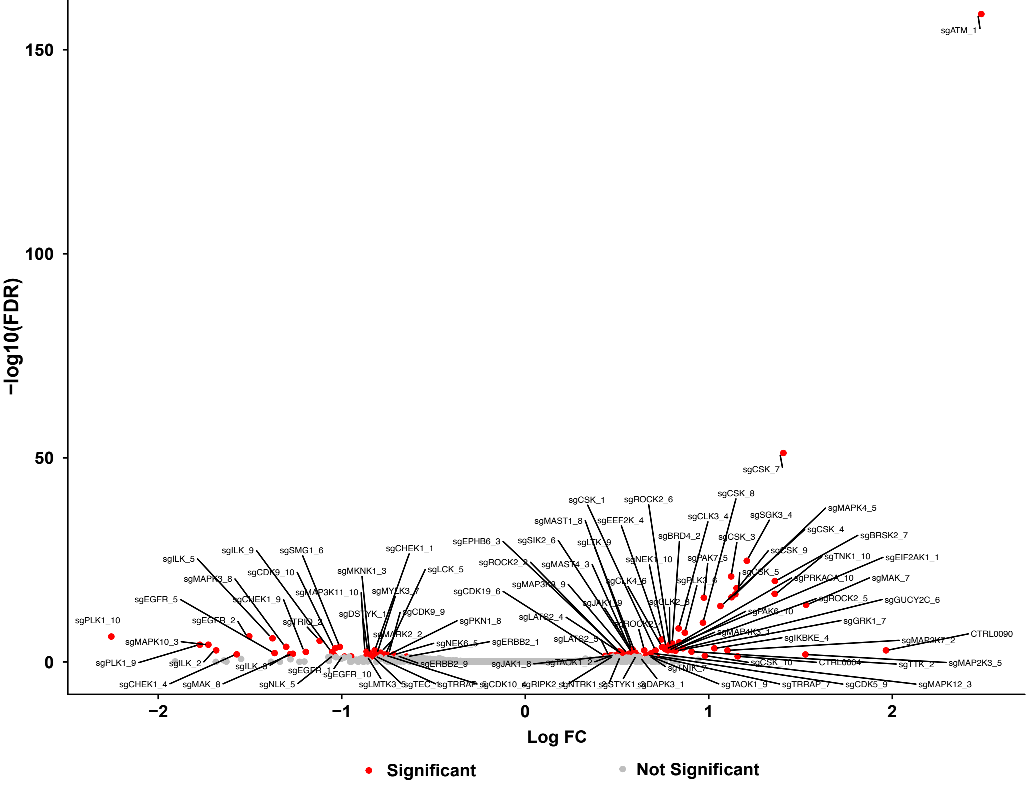

Figure S5. The negatively and positively enriched sgRNAs upon FGFR inhibition. Significance (FDR<0.5) were calculated by MAGeCK. Significant sgRNAs were labeled and displayed as red dots. FC – Fold Change.

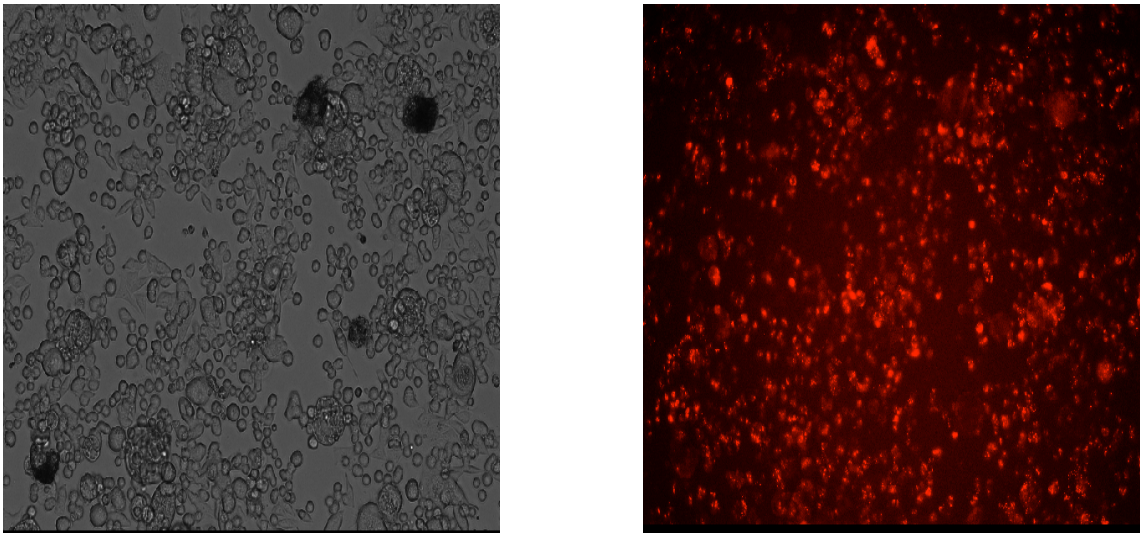

Figure S6. High siRNA transfection efficiency in KatoIII cells. Kato III cells were seeded in 6 well plates at 300,000 cells/well the day before transfection and were transfected with 10 nM Cy 3 Control DsiRNA oligos (IDT) using LipoJet.

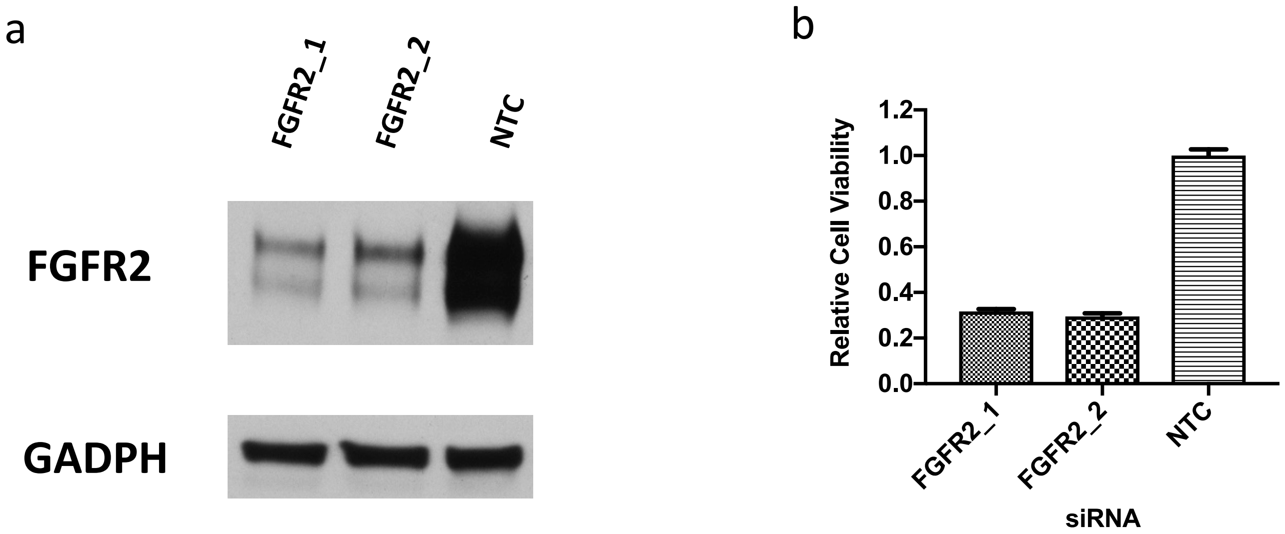

Figure S7. FGFR2 knockdown using siRNAs in KatoIII cells. (a) Western blot confirmed that FGFR2 gene expression was drastically reduced in KatoIII cells. Cells were transfected with 10 nmol/l oligos and were harvested 72 h. GADPH was the loading control. (b) Knocking down FGFR2 expression alone notably reduced KatoIII cell viability. The Dicer-substrate short interfering RNAs (DsiRNAs) targeting FGFR2 (FGFR2_1 Reference #207585853 and FGFR2_2 #207585856) as well Negative control oligos (NTC) were obtained from IDT. The viability was measured six days by CellTiter-Glo. Representative data from one of three independent experiments are shown.

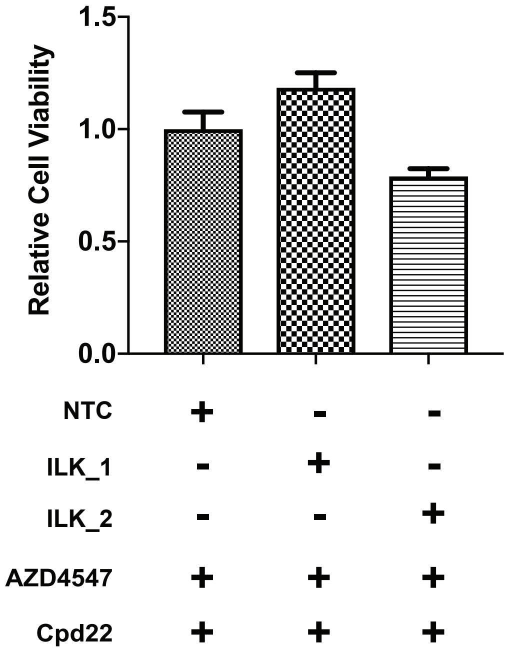

Figure S8. The effects of Cpd22 in combination with AZD4547 on KatoIII cells with ILK knockdown. Cpd22 drastically reduced the effects of ILK knockdown on AZD4547 treated KatoIII cells. The viability was measured six days by CellTiter-Glo.

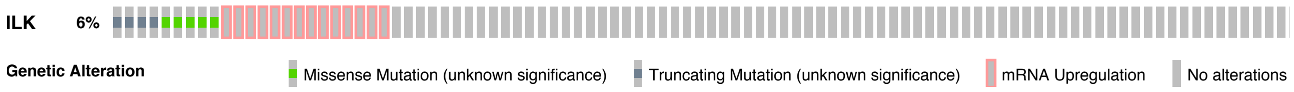

Figure S9. Mutational status of ILK in gastric cancer. ILK was altered in 6% (25 of 415) TCGA gastric tumors. The mutational status was displayed in the OncoPrint by cBioportal .
